# Behavior Study of Commercial Polyurea under Monotonic, Rate Dependent, Cyclic, and Fatigue Tensile Loading for Potential Structural Applications

**DOI:** 10.3390/polym14091878

**Published:** 2022-05-04

**Authors:** Pawan Acharya, Hamed Ebrahimian, Mohamed A. Moustafa

**Affiliations:** Department of Civil and Environmental Engineering, University of Nevada Reno, Reno, NV 89557, USA; pacharya@nevada.unr.edu (P.A.); mmoustafa@unr.edu (M.A.M.)

**Keywords:** polyurea, tensile loading, rate dependent loading, cyclic loading, fatigue loading, structural polyurea

## Abstract

Understanding material behavior is key to discovering innovative applications in any field. Regardless of the exciting mechanical properties of polyurea, there has been a limited effort in studying the use of polyurea for structural retrofit and strengthening applications. This study aims to understand the behavior of polyurea under different tensile loading conditions to provide critical information towards enabling the future use of polyurea in structural applications. Several standard coupons are tested under various tensile loading conditions to understand the mechanical behavior of eight different commercial polyureas. The study provides the full stress–strain characteristic curves that can be used for constitutive modeling purposes. The results show that polyurea has a wide range of properties from low strength flexible nature to high strength rigid nature. All tested polyureas displayed some level of rate dependency, i.e., ultimate strength is a function of loading rates. The high-strength polyureas tested only show slight rate dependency and good strength retention under cyclic and fatigue tensile loading, suggesting that polyureas have promising mechanical properties for potential structural applications.

## 1. Introduction

The civil infrastructure across the United States is aging and deteriorating. The demolition and replacement of infrastructures demands a massive investment of time and money. Thus, the repair and retrofit of existing structures are preferred to extend their service life. Significant research efforts are pursued to explore new materials and methods for reinforced concrete strengthening or retrofitting, e.g., the application of fiber reinforced polymers and glass fiber reinforced polymers wraps, steel plates [1], and steel jacketing [2]. Although these methods are highly effective, their application is costly and time-consuming. Thus, this research aims to explore the application of polyurea for structural strengthening purposes.

**Figure 1:**
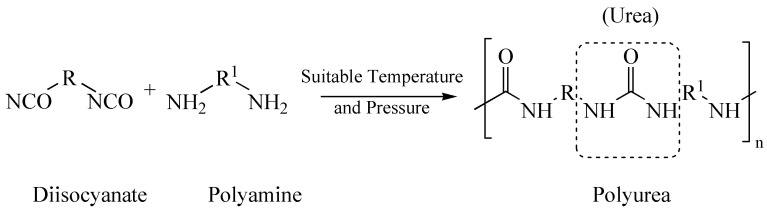
Polyurea formation chemical reaction. R and R1 denote alkyl groups.

Polyurea is a type of elastic polymer formed by the polymerization reaction of two components, polyamine and diisocyanate, as shown in Figure 1. The polyurea has a chain-type structure that accounts for its flexible nature and provides the ability to form a durable coating over a surface. The polyurea application on a surface includes three general steps: cleaning the surface, mixing components, and spray coating. Initially, the surface to be coated is thoroughly cleaned and dried to bond the coating to the surface properly. For a typical application, a pneumatic spraying device mixes the two polyurea components at the required temperature and pressure and sprays the mixture in different layers perpendicular to one another [3].

Polyurea coating has essential applications for blast resistance and shock proofing in military defense as the material can absorb and dissipate high-rate energy [4,5,6]. The material has high durability, chemical resistance, and weathering resistance, making it suitable for numerous applications in the coating industry that include aesthetics, hydro isolation, leak proofing of chemicals, and corrosion prevention. Due to its good abrasion resistance, it is used in industrial flooring and parking lots enduring heavy moving loads. The strong adhesion property of polyurea enables the confinement of spalled concrete fragments under regular [7,8,9] and blast loading [4].

Several experimental studies have been conducted to understand the behavior of polyurea under different loading conditions, establish their mechanical properties, and develop predictive numerical models. Beyer et al. [7] assessed the compressive strength, stiffness, and ductility of polyurea sprayed concrete cylinders subjected to accelerated environmental conditions. Standard cylindrical concrete samples were tested for axial compressive strength after being subjected to freeze and thaw cycles and deicing chemical attacks. The results showed that polyurea coating provides good durability, adhesion, and enhances ductility. Yi et al. [10] studied the rate-dependent stress–strain behavior of the polyurea and polyurethanes under compression in strain rate loading ranging from 10^3^/s to 10^4^/s. The polyurea showed a behavioral transition from a rubber-like material at lower strain rates (10^−3^/s–10/s) to leather-like material behavior at high strain rates (~10^3^/s). Similar conclusions about the strain rate dependency due to the transition of the polyurea material from rubbery to leathery phase under uniaxial compressive and tensile loadings were provided by Sarva et al. [11], Raman et al. [12], and Wang et al. [13]. Holzworth [14] prepared seven polyurea material types by changing the ratio of isocyanate to amine from 95% to 120% and tested them to determine the change in glass transition temperature and mechanical properties. The results showed that the increase in the isocyanate content could increase the stiffness of the polyurea material. Several material constitutive models of polyurea have been proposed by Mohotti et al. [15], Wang et al. [13], and Guo et al. [16] to define the tensile behavior of polyurea at high strain rates. Nevertheless, there is limited knowledge in the literature on the properties of commercially available polyurea for loading rates that are pertinent to civil and structural applications.

A large body of literature is focused on studying the polyurea behavior under blast and impact loads and its applications. Davidson et al. [4] studied the blast performance of polyurea-retrofitted masonry walls. The explosive testing results showed the remarkable effects of polyurea as a retrofit coating to improve the confinement of blast fragments for masonry walls. Hrynyk and Myers [17] investigated the out-of-plane behavior of unreinforced masonry arching walls reinforced with polyurea and glass fiber reinforced polymer (GFRP). The masonry walls showed a brittle failure under out-of-plane blast loading. However, the use of polyurea retrofit increased the out-of-plane loading and energy dissipation capacities of unreinforced masonry and reduced or prevented the masonry debris scatter upon collapse. The polyurea coating application as a reinforcement for different constructions, including reinforced concrete panels [18], steel plates [19], high-performance cementitious composites [20], and as a helmet suspension pad material [5], have been investigated under blast or impact loadings. Collectively these studies presented polyurea as an excellent material for blast retrofit applications.

There has been limited literature on the structural application of polyurea under regular loading conditions. Greene et al. [21] performed flexural and shear tests on eight beams specimens sprayed with polyurea with/without chopped E-glass fibers. A beam with 6 mm polyurea coating showed a 23.8% increase in ultimate beam capacity in flexure. Marwan et al. [22] investigated a total of sixteen reinforced concrete beams strengthened with polyurea coating (ten in shear and six in flexure). Results showed an increase in flexural capacity by 19.4% in shorter beams and 11.2% in longer beams for 6 mm thick polyurea spray. Additional shear capacity of 42.5% in shorter beams and 28.2% in longer beams were added by the 6 mm thick polyurea coating. Parniani and Toutanji [23] examined the behavior of RC beams sprayed with polyurea under monotonic loading (1.5 mm/min) and fatigue loading (sinusoidal loading with a load ratio of 0.2). Two thicknesses of polyurea coating, 2.5 mm and 5 mm, were used. Results showed an increase in flexural capacity by 9.2% and 17.2% for 2.5 mm and 5 mm thick polyurea spray, respectively. The coating improved the ductility of the beams. A theoretical model for behavior of polyurea strengthened beams under cyclic non-reversal flexural loading was proposed that showed satisfactory results for midspan load-deflection for up to one million loading cycles. In other studies by Greene and Myers [21], Carey and Myers [24], and Song et al. [25], polyurea coating integrated with glass fibers showed a significant increase in flexural, shear, and ductility of concrete members. Studies of sprayed polyurea on tensile strength of concrete slabs by Rizwan et al. [26], crushing strength of concrete rings by Szafran and Matusiak [27], and compressive strength of columns by Tuhin and Tazarv [8] showed that polyurea could aid in improving the strength of the concrete members.

Despite the significant body of related literature as summarized above, there is still a major knowledge gap that this study will contribute to. There is a lack of full comprehensive tensile stress–strain relationships for the wide range of readily and commercially available polyurea under different tensile loading scenarios such as rate-dependent or cyclic loading and unloading. Such fundamental and full stress–strain behavior is inevitable for constitutive modeling, sectional analysis, and structural design for repair and retrofit applications. Meanwhile, most of the previous work that focused on structural applications did not provide the full material characterization of the adopted retrofit solution, which hinders the expansion of those studies towards developing and calibrating analytical models or design guidance. Therefore, the main objective of this study is to understand and document the full stress–strain tensile behavior under different loading scenarios of a wide range of polyurea with varying strength and strain capacities. This research can provide a catalog of different types of polyurea that can be used as first step to examine and investigate the innovative structural applications of elastomeric polymers. The objective here is to compare the tensile properties of commercial polyurea. For this purpose, monotonic, rate-dependent, cyclic, and fatigue tensile tests are performed on a total of eight types of commercial polyurea material coupons to evaluate and compare their tensile behavior. Based on the discussed test results, we conclude the study by providing recommendations on the polyurea material with promising mechanical properties for further research on structural strengthening applications.

## 2. Materials and Methods

### 2.1. Polyurea Material

Eight different types of commercial polyurea materials are used in this study. Table 1 shows the list of polyurea materials with the commercial names, assigned material type, and the characteristics according to the manufacturer datasheets [28,29,30,31,32,33,34]. Gel time is the time for polyurea to become highly viscous and non-workable. Tack-free time is the time the polyurea material surface becomes non-sticky.

### 2.2. Coupon Preparation and Test Setup

**Figure 2:**
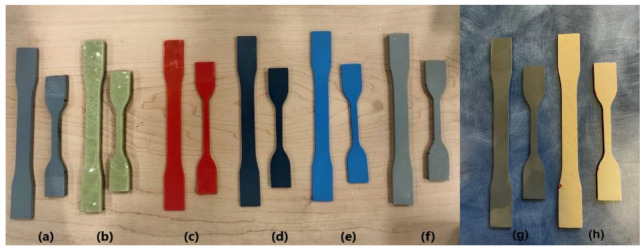
Coupon samples of Polyurea materials (**a**) S1, (**b**) S2, (**c**) S3, (**d**) S4, (**e**) S5, (**f**) S6, (**g**) S7, and (**h**) S8.

Test coupons were cut out from carefully prepared polyurea sheets of various dimensions. The coupons for testing were cut into two ASTM D638 [35] die types: die type I and die type IV. Figure 2 shows a sample of the various coupon specimens cut out for the tests. Test coupons of S3, S4, S5, S6, and S8 polyurea materials were prepared manually using dies and a 1-ton Arbor press. However, S1, S2 and S7 polyurea materials were too stiff for manual cutting. Therefore, the test coupons were cut from sheets using a water jet device. Since the thickness of the coupons was nonuniform, we measured and averaged the thickness of coupons at the two ends and the middle.

**Figure 3:**
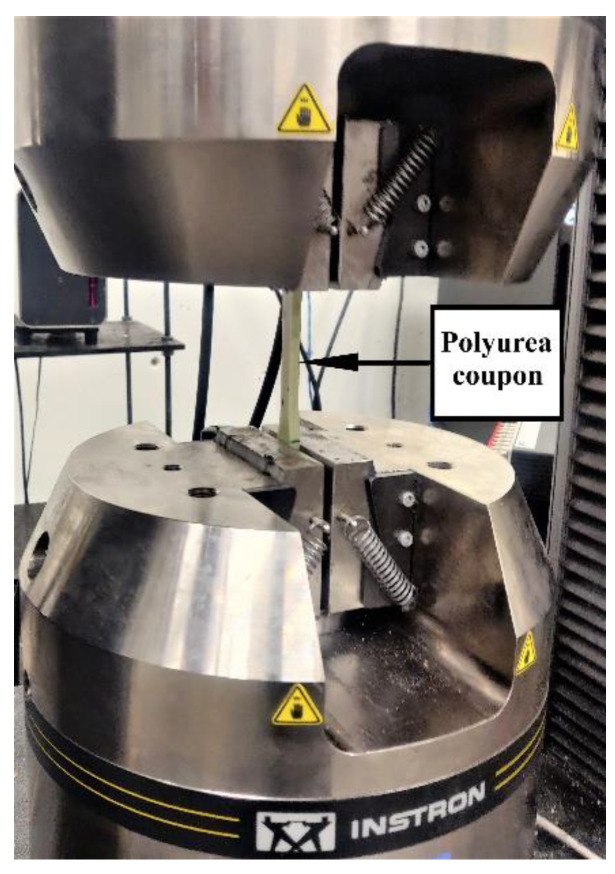
Test Setup featuring a typical die type I coupon specimen installed in the Instron UTM.

Different type of tensile tests that include monotonic, rate-dependent, cyclic, and fatigue tests were performed to understand the mechanical behavior of polyurea materials. All tests were performed using a 200-kN Instron^®^ universal testing machine (UTM) that is equipped with high-performance hydraulic grips for the proper testing of specimens with various thicknesses. Coupon samples were placed in the UTM maintaining consistent grip spacing. The inbuilt transducers in the Instron UTM recorded load and displacement data. Figure 3 shows the test setup. Both die-type coupons for all polyurea material types were tested for monotonic and rate dependent tensile tests. The die type I coupons of S1 and S7 materials were further tested for cyclic and fatigue tensile tests for full characterization, as they showed promising strength properties for potential structural applications.

### 2.3. Monotonic Tensile Tests

Uniaxial monotonic tension tests were performed for both ASTM D638 [35] die type I and IV polyurea coupons. Loading rates for the coupons were evaluated based on the ASTM recommendation. Four coupons for each die size were tested for the each polyurea type. Since the stress–strain response behavior was observed to be consistent among the four samples, only two coupons for the other die types were tested. Test matrix and loading rates used for each polyurea material with the average thickness and number of coupons tested are shown in Table 2.

### 2.4. Rate Dependent Tensile Tests

The coupon samples of polyurea were tested under three different loading rates selected to represent typical structural applications, such as retrofitting for vehicular impacts and seismic loading events. From the literature, strain rates in structures for vehicular impact range from 10^−4^/s to 10^−3^/s [36] and for earthquake ground motions range from 10^−3^/s to 10^−1^/s, as observed by Li et al. [37]. This information, combined with the measured grip spacing compatible for test coupons, was used to estimate appropriate loading rates according to the following relation (Equation (1)), where grip spacing corresponds to the original length of the test coupons.

Loading Rate (in/min^−1^) = Strain Rate (min^−1^) × Grip Spacing (in) (1)

Based on the loading rates obtained using the strain rates from the literature quoted above, the minimum loading rate chosen for rate-dependent tensile testing was 0.2 in/min (5.08 mm/min), the maximum loading rate was chosen to be 20 in/min (508 mm/min, maximum loading rate capacity of the equipment), and the mean loading rate was selected to be 10 in/min (254 mm/min).

The coupons used, test setup, and test procedure were similar to the standard ASTM monotonic tensile tests. Table 3 shows the test matrix for the rate-dependent tensile tests.

### 2.5. Cyclic Tensile Test

**Figure 4:**
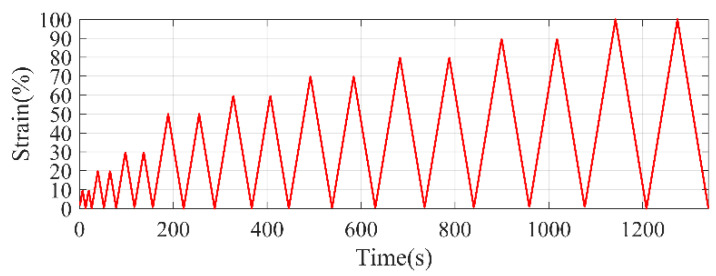
Loading pattern for cyclic test.

Die type I coupons of S1 and S7 polyurea materials were tested under non-reversal cyclic tensile loading. The coupons, test setup, and data acquisition were similar to the tensile tests previously discussed. The loading rate used for the test is 5.08 mm/min (0.2 in/min). Three coupons for each polyurea material were tested under cyclic tensile loading with a loading pattern as shown in Figure 4.

### 2.6. Tensile Fatigue Test

Die type I coupons of S1 and S7 materials were tested for tensile fatigue loading. The fatigue load used in this test is 70% and 90% of the average peak loading from the tensile tests. The samples were loaded either up to 150 cycles or until the failure for each fatigue load case. The test setup was again similar to the tensile test setup shown before in Figure 3. The loading rate was 50.8 mm/min (2 in/min) for all polyurea coupons.

## 3. Results and Discussions

### 3.1. Tensile Behavior of Different Material Types

**Figure 5:**
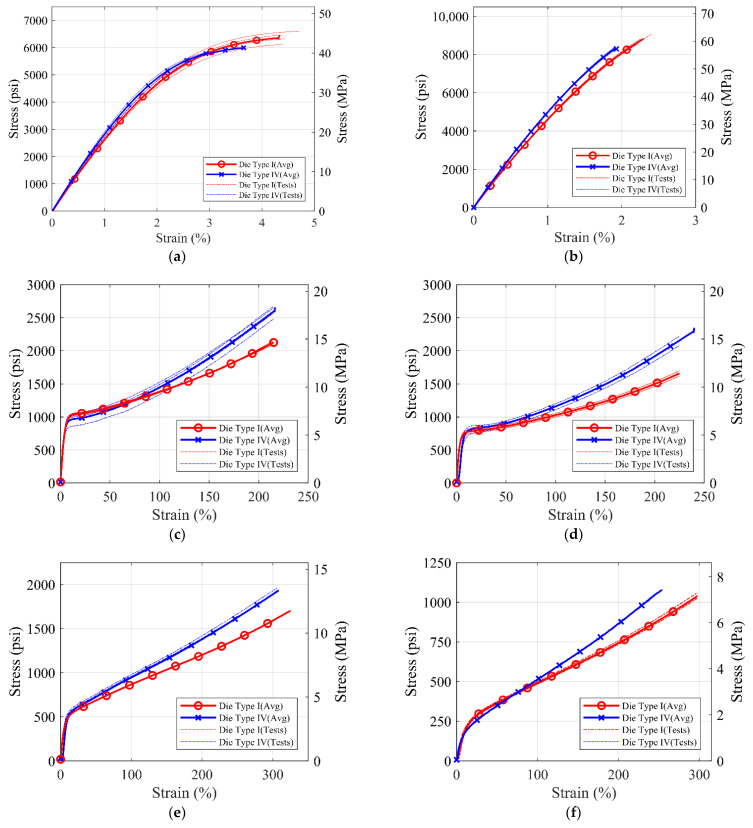

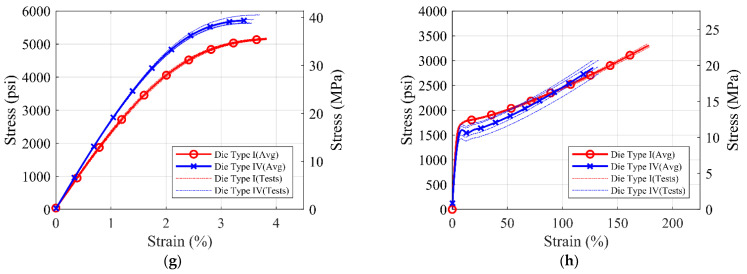
Stress-strain curves for (**a**) S1, (**b**) S2, (**c**) S3, (**d**) S4, (**e**) S5, (**f**) S6, (**g**) S7, and (**h**) S8 material for ASTM D638 Die Type I and Die Type IV coupons for monotonic tensile loading.

Load and displacement data acquired by the Instron UTM are post-processed to get stress–strain values for the tests. Engineering stress values are calculated by dividing the load values by the initial coupon sectional area at the narrow section, i.e., desired failure location. Engineering strain values are obtained by dividing the extension values to the original grip spacing measured at the start of the test. Figure 5 shows the average tensile stress–strain plot generated by averaging the stress values at constant strain values from all tested coupons for different die types and polyurea material types.

Table 4 summarizes the yield strength, elongation, and ultimate strength of different polyurea. The yield strength is calculated using the 0.2% strain offset method [38]. The slope of the linear portion of the stress–strain curve is calculated to obtain the initial stiffness of the polyurea material.

### 3.2. Tensile Behavior Comparison of Different Material Types

**Figure 6:**
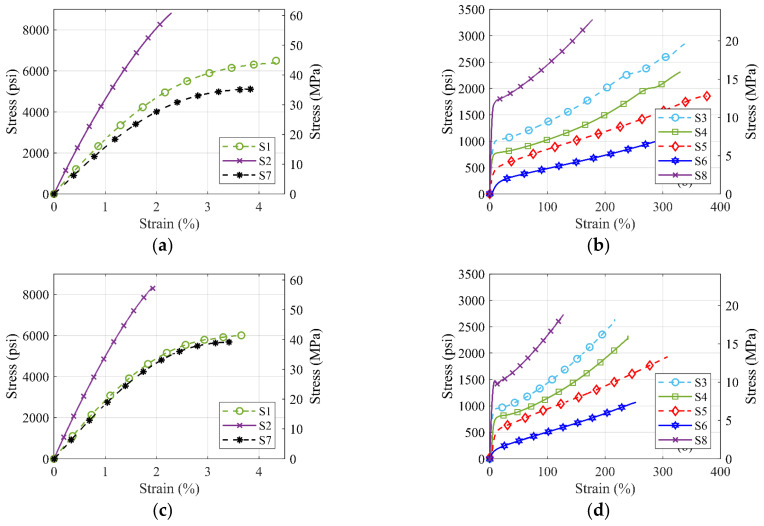
Comparison of monotonic tensile stress-strain response of (**a**) ASTM die type I coupons of S1, S2, and S7 material, (**b**) ASTM die type I coupons of S3, S4, S5, S6, and S8 material, (**c**) ASTM die type IV coupons of S1, S2, and S7 material, (**d**) ASTM die type IV of S3, S4, S5, S6, and S8 material.

It can be observed from Figure 5 and Table 4 above that the ultimate stress values for S1, S2, and S7 materials are higher than S3, S4, S5, S6, and S8 polyurea. However, the rupture strain of S1, S2, and S7 materials is less than 4%, while the rupture strain of S3, S4, S5, and S6 exceeds 100%. Figure 6 shows the combined average stress–strain curves from monotonic tensile tests for different commercial polyureas. Figure 6a,c shows a comparison for the high-strength S1, S2, and S7 polyureas, while Figure 6b,d compares the low-strength S3, S4, S5, S6, and S8 polyureas for die types I and IV, respectively.

The strongest pure polyurea material is the S2 material with a maximum tensile strength of 43.4 MPa (6.3 ksi), and S6 is the weakest material with a maximum strength of 6.6 MPa (0.96 ksi). S2 polyurea has the highest strength of 43.4 MPa (6.3 ksi) but the lowest ductility with a rupture strain of only 1.92%. S5 polyurea has the lowest strength of 6.6 MPa (0.96 ksi) but the highest ductility with a rupture strain of 376%. A trend of ductility loss with increased strength and initial stiffness can be seen in the comparative results of the tested commercial polyureas. Moreover, these results confirm that S1, S2, and S7 materials have high ultimate strength and larger initial stiffness and could be suitable for structural applications.

Upon comparing the test results of standard coupons (die type I) of S1 and S2 materials, the tensile strength of S2 material 60.7 MPa (8.8 ksi) is 1.4 times greater than that of S1 material 43.4 MPa (6.3 ksi). However, the strain at rupture decreases to 1.92% from 2.99%. So, it is observed that the addition of glass fibers into the polyurea matrix increases the material strength but reduces the ductility.

### 3.3. Rate Dependent Behavior of Different Material Types

**Figure 7:**
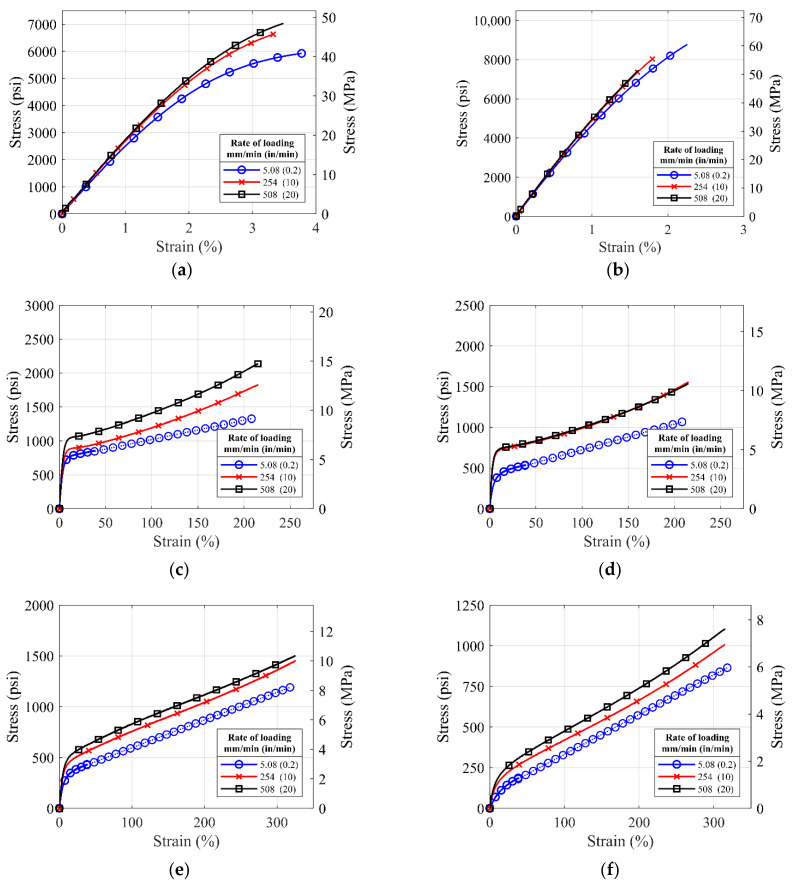

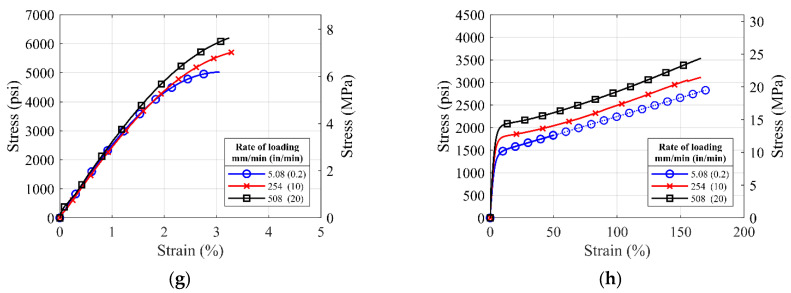
Average stress-strain curves for ASTM die type I coupons of (**a**) S1, (**b**) S2, (**c**) S3, (**d**) S4, (**e**) S5, (**f**) S6, (**g**) S7, and (**h**) S8 material tested with loading rates of 0.2 in/min, 10 in/min and 20 in/min.

**Figure 8:**
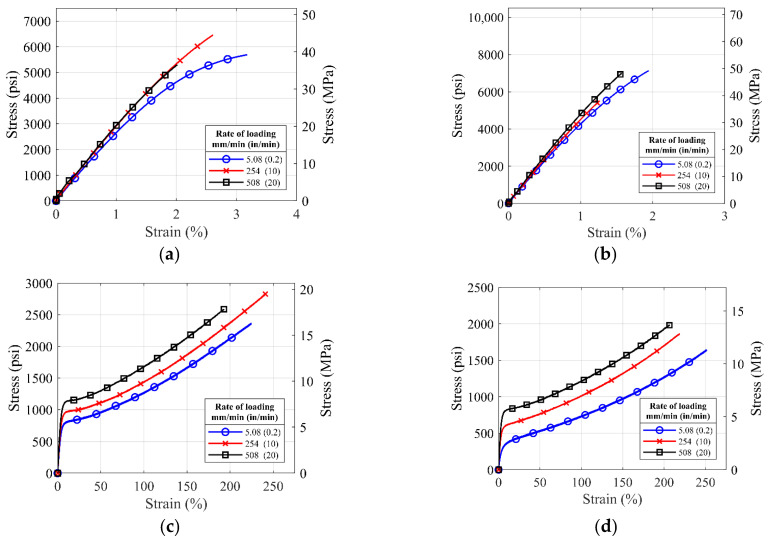

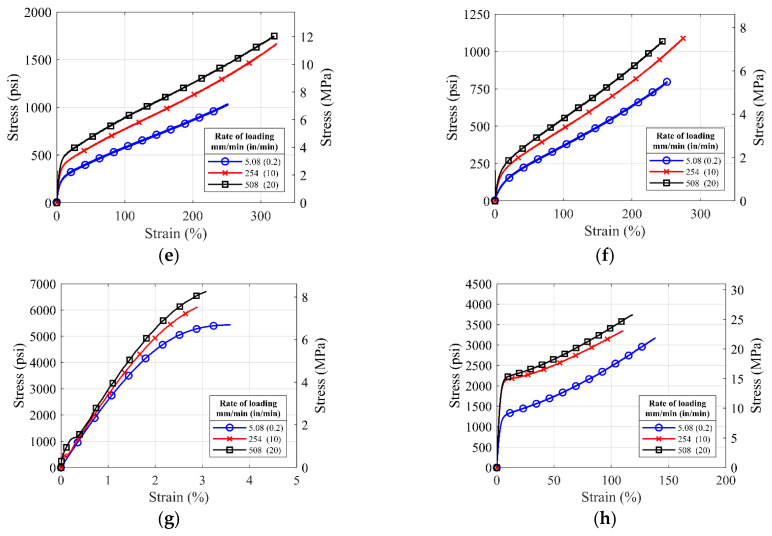
Average stress-strain curves for ASTM die type IV coupons of (**a**) S1, (**b**) S2, (**c**) S3, (**d**) S4, (**e**) S5, (**f**) S6, (**g**) S7, and (**h**) S8 material tested with loading rates of 0.2 in/min, 10 in/min and 20 in/min.

Figure 7 and Figure 8 show the average stress–strain curves generated from the rate-dependent tests on the different commercial polyurea. For the loading rate of 0.2 in/min and die type I coupons of S3, S4, S5, and S6 materials, test to failure encountered grip slippage problems and long test time. So, the tests were terminated at the elongation of 2 in. The dotted lines are the projected estimate for the tests terminated.

Based on Figure 7 and Figure 8, it is noted that the polyurea materials show some degree of rate dependency. For all pure polyurea materials (S1–S8 except S2), the strength of the polyurea increases with the increasing rate of loading, a trend commonly observed in viscoelastic materials. The degree of the rate dependency is prominent in the S3, S4, S5, S6, and S8 materials, while S1 and S7 materials show low rate dependency because of their high stiffness. However, rate dependency for the S2 polyurea is not distinct compared to other polyureas, which might have resulted from the addition of the glass fibers to the polyurea matrix that increased material stiffness. The S1, S2, and S7 materials have been shown to have high tensile strength and insignificant rate-dependent behavior.

### 3.4. Cyclic Tensile Behavior of Selected Material Types

**Figure 9:**
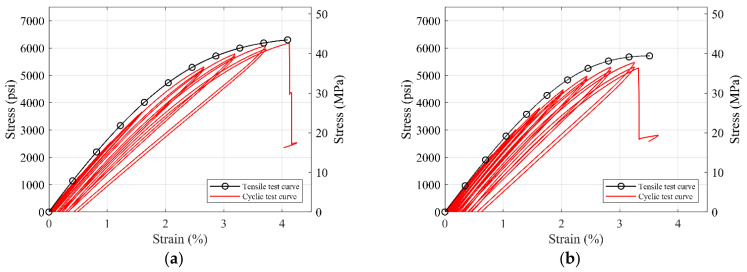
Average stress-strain curve for the cyclic tensile test of (**a**) S1 and (**b**) S7 material.

As the pure polyurea materials S1 and S7 showed desirable mechanical properties in the monotonic and rate-dependent tensile test, they were rendered as the most promising for potential structural applications, and in turn, were tested further under cyclic and fatigue loading. Figure 9 shows the average stress–strain curve generated from the cyclic tensile tests of the S1 and S7 polyurea.

It is observed that the unloading curve of polyurea nearly overlaps the reloading curve until the third cycle of loading. There is no distinct softening effect due to the cyclic loading. After the polyurea coupon yields, the unloading and reloading curves get separated, and plastic deformation is observed. The unloading stiffness for both materials decreases gradually. This indicates the nonlinear behavior of the polyurea material. The backbone curve of the cyclic test is consistent with the average tensile test’s stress–strain curve showing only a slight decrease in strength under the cyclic loading as the material enters the nonlinear phase. These results add to the suitable mechanical properties of polyurea material for wide range of structural applications.

### 3.5. Tensile Fatigue Behavior of Selected Material Types

**Figure 10:**
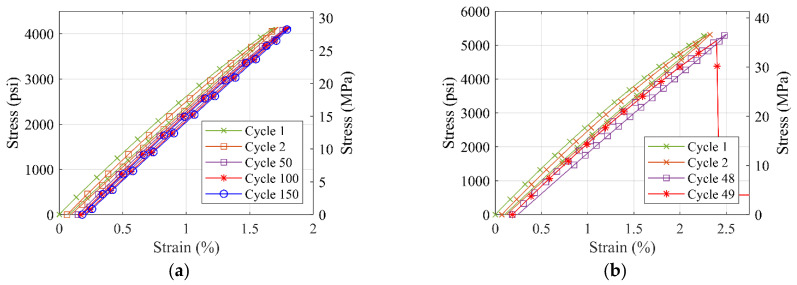

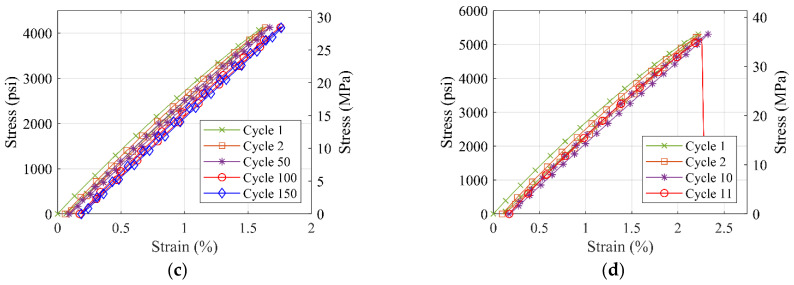
Stress-strain curve for tensile fatigue testing of S1 material with (**a**) 70% of the max load for sample 1 (**b**) 90% of the max load for sample 1 (**c**) 70% of the max load for sample 2 (**d**) 90% of the max load for sample 2.

**Figure 11:**
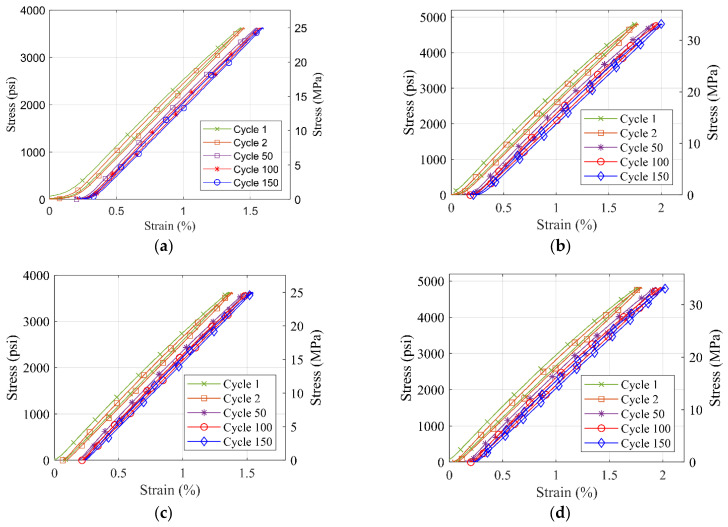
Stress-strain curve for fatigue testing of S7 material with (**a**) 70% of the max load for sample 1 (**b**) 90% of the max load for sample 1 (**c**) 70% of the max load for sample 2 (**d**) 90% load for sample 2.

S1 and S7 material coupons are tested to failure or complete 150 cycles under 70% and 90% of the max loading obtained from tension tests results. Both S1 and S7 material coupons endured 150 cycles for 70% of the max load. However, S1 material coupons failed at the 11th and 48th cycles for 90% of the max load. As for S7 material, both coupons endured the complete 150 cycles for 90% of max load. Figure 10 and Figure 11 show the stress–strain curves for the tensile fatigue testing for both load ratios of the two materials.

For both tested polyurea material types, it is observed that the slope of the stress–strain curve does not seem to change drastically, but a small plastic deformation is observed in each cycle of the fatigue loading that accumulates with every cycle. These results suggest that a further in-depth study of the fatigue load behavior for the polyurea material is needed, which is suggested for future work.

## 4. Conclusions

The study investigated the stress–strain behavior of commercial polyureas under various tensile loading scenarios, such as monotonic loading, varying rate of loading, cyclic loading, and fatigue loading. The results from the tensile monotonic tests showed that polyurea could vary from high strength to low strength with an ultimate tensile capacity of 43.4 MPa (6.3 ksi) and 6.62 MPa (0.96 ksi), respectively. Similarly, a wide variation in ultimate strain can be observed that ranged from 2.99% to more than 376%. From the rate dependency tests, it was seen that the S1 (Arostruct^®^), S2 (Arostruct^®^ with fibers), and S7 (Nukote PP300) polyurea material types show insignificant rate-dependent behavior on the loading rates equivalent to the strain rates due to earthquake loading. The rate-dependent tests confirm an increase in the ultimate strength of polyurea with the increase in the rate of loading, but with a decrease in ductility capacity. The cyclic loading tests for S1 (Aro-Struct) and S7 (Nukote PP300) material show only a slight decline in strength, even under repeated cyclic loading. These results concluded that S1 material (Arostruct^®^) and S7 (Nukote PP300) polyurea types have the most promising mechanical properties and are thus the class of polyureas recommended for structural applications.

The findings showed an increase in ultimate strength from S1 (Arostruct^®^) to S2 (Arostruct^®^ with fibers) material due to the addition of glass fibers. So, it is proposed that the addition of fibers might improve the polyurea properties for structural purposes. Accordingly, more research is recommended for future studies to understand the behavior of polyurea mixed with different type of fibers and the effect of fibers on the mechanical properties of the composite material.

In closing, this study provides a comprehensive assessment of the tensile properties of a wide range of polyureas under different loading scenarios. However, a key contribution of this study is providing, for the first time, full stress–strain characterization and relationships for this large range of polyureas, which can be readily used for constitutive modeling and analytical investigations of future structural applications of polyurea.

## Figures and Tables

**Table 1:** Properties of polyurea used in the study based on the manufacturer’s datasheet.

Material Type (S#)	Commercial Name	Rupture Strength MPa (psi)	Elongation at Rupture	Gel Time	Tack Free Time
S1	Arostruct^®^	41.4 (6000)	2.5–3.5%	10–12 s	2 min
S2	Arostruct^®^ with fibers *	n/a	n/a	n/a	n/a
S3	Bridge Deck Membrane	13.8 (2000)	>250%	10 s	30 s
S4	Bridge Deck Membrane Topcoat	13.8 (2000)	>250%	90 s	2 min
S5	AquaVers^TM^-405	15.2 (2200)	>500%	25 s	180 s
S6	SL/75^TM^	8.9 (1295)	>406%	1 min	10 min
S7	Nukote PP300	55 ± 3 (8000 ± 500)	2 ± 1%	15–20 s	60–90 s
S8	Nukote HTD	26 ± 2 (3550 ± 250)	200–300%	5–15 s	30–45 s

* The information on the properties of the material was not disclosed by the manufacturer.

**Table 2:** Test matrix, loading rates used, polyurea coupon thickness, and number of coupons tested.

Material Type	Die Type	Loading Rate Used mm/min (in/min)	Average Coupon Thickness mm (in)	Number of Coupons Tested	
				Die Type I	Die Type IV
S1	I, IV	5.08 (0.2)	5 (0.197)	4	2
S2		5.08 (0.2)	6 (0.236)	4	2
S3		254 (10)	2 (0.079)	2	4
S4		254 (10)	2 (0.079)	2	4
S5		254 (10)	2 (0.079)	2	4
S6		254 (10)	4 (0.157)	4	2
S7		5.08 (0.2)	2.5 (0.098)	2	4
S8		254 (10)	2 (0.079)	2	4

**Table 3:** Test matrix for the rate-dependent test.

Material Type	Die Types	Loading Rates Used mm/min (in/min)	Average Coupon Thickness mm (in)	Number of Coupons Tested	
				Die Type I	Die Type IV
S1	I, IV	5.08 (0.2) 254 (10) 508 (20)	5 (0.197)	2	2
S2			6 (0.236)	2	2
S3			2 (0.079)	2	2
S4			2 (0.079)	2	2
S5			2 (0.079)	2	2
S6			4 (0.157)	2	2
S7			2.5 (0.098)	2	2
S8			2 (0.079)	2	2

**Table 4:** Tensile tests results summary for eight different tested commercial polyureas.

Polyurea Material	ASTM D638 Die Type	0.2% Offset Yield Strength MPa (ksi)	Initial Stiffness MPa (ksi)	Rupture Strength MPa (ksi)	Strain at Rupture (%)
S1	Die Type I	27.7 (4.02)	1903 (276)	43.4 (6.29)	4.09
	Die Type IV	28.5 (4.14)	2013 (292)	40.1 (5.81)	2.99
S2	Die Type I	52.5 (7.61)	3199 (464)	60.7 (8.80)	2.29
	Die Type IV	54.6 (7.92)	3454 (501)	57.9 (8.40)	1.92
S3	Die Type I	6.0 (0.87)	110 (16)	19.3 (2.79)	>339 *
	Die Type IV	6.1 (0.88)	110 (16)	17.9 (2.59)	>216 *
S4	Die Type I	4.3 (0.63)	110 (16)	15.9 (2.30)	>331 *
	Die Type IV	4.6 (0.66)	103 (15)	15.9 (2.30)	>339 *
S5	Die Type I	2.8 (0.41)	48 (7)	11.7 (1.70)	>376 *
	Die Type IV	2.5 (0.36)	48 (7)	12.4 (1.80)	>284 *
S6	Die Type I	1.0 (0.15)	14 (2)	6.6 (0.96)	>274 *
	Die Type IV	1.1 (0.16)	14 (2)	7.6 (1.10)	>248 *
S7	Die Type I	25.65 (3.72)	1689 (245)	36.34 (5.27)	4.03
	Die Type IV	23.99 (3.48)	1827 (265)	39.44 (5.72)	3.51
S8	Die Type I	10.5 (1.53)	310 (45)	23.9 (3.46)	179
	Die Type IV	9.8 (1.42)	296 (43)	21.9 (3.17)	137

* The strain at rupture values for S3, S4, S5, and S6 material are approximate as the grip slippage occurred before the coupons ruptured.

## Data Availability

All data or code that support the findings of this study are available from the corresponding author upon reasonable request.

## References

[1] Ghaffary A., Moustafa M.A. (2020). Synthesis of Repair Materials and Methods for Reinforced Concrete and Prestressed Bridge Girders. Materials.

[2] Wu Y.F., Liu T., Oehlers D.J. (2016). Fundamental Principles That Govern Retrofitting of Reinforced Concrete Columns by Steel and FRP Jacketing. Adv. Struct. Eng..

[3] Szafran J., Matusiak A. (2016). Polyurea Coating Systems: Definition, Research, Applications.

[4] Davidson J.S., Porter J.R., Dinan R.J., Hammons M.I., Connell J.D. (2004). Explosive Testing of Polymer Retrofit Masonry Walls. J. Perform. Constr. Facil..

[5] Grujicic M., Bell W.C., Pandurangan B., He T. (2010). Blast-Wave Impact-Mitigation Capability of Polyurea When Used as Helmet Suspension-Pad Material. Mater. Des..

[6] Iqbal N., Sharma P.K., Kumar D., Roy P.K. (2018). Protective Polyurea Coatings for Enhanced Blast Survivability of Concrete. Constr. Build. Mater..

[7] Beyer M.E., Myers P.J.J. Durability Performance of Polyurea Based Systems for Concrete Member Rehabilitation. https://citeseerx.ist.psu.edu/viewdoc/summary?doi=10.1.1.689.4970.

[8] Tuhin I., Tazarv M. (2020). Stress-Strain Relationship for Polyurea-Confined Circular Concrete Columns under Static Loads. ACI Mater. J..

[9] Akın E., Tunaboyu O., Avşar Ö. (2020). Axial Behavior of FRP Confined Low-Strength Concrete with Polyurea. Structures.

[10] Yi J., Boyce M.C., Lee G.F., Balizer E. (2006). Large Deformation Rate-Dependent Stress-Strain Behavior of Polyurea and Polyurethanes. Polymer.

[11] Sarva S.S., Deschanel S., Boyce M.C., Chen W. (2007). Stress–Strain Behavior of a Polyurea and a Polyurethane from Low to High Strain Rates. Polymer.

[12] Raman S.N., Ngo T., Lu J., Mendis P. (2013). Experimental Investigation on the Tensile Behavior of Polyurea at High Strain Rates. Mater. Des..

[13] Wang H., Deng X., Wu H., Pi A., Li J., Huang F. (2019). Investigating the Dynamic Mechanical Behaviors of Polyurea through Experimentation and Modeling. Def. Technol..

[14] Holzworth K., Jia Z., Amirkhizi A.V., Qiao J., Nemat-Nasser S. (2013). Effect of Isocyanate Content on Thermal and Mechanical Properties of Polyurea. Polymer.

[15] Mohotti D., Ali M., Ngo T., Lu J., Mendis P. (2014). Strain Rate Dependent Constitutive Model for Predicting the Material Behaviour of Polyurea under High Strain Rate Tensile Loading. Mater. Des..

[16] Guo H., Guo W., Amirkhizi A.V., Zou R., Yuan K. (2016). Experimental Investigation and Modeling of Mechanical Behaviors of Polyurea over Wide Ranges of Strain Rates and Temperatures. Polym. Test..

[17] Hrynyk T.D., Myers J.J. (2008). Out-of-Plane Behavior of URM Arching Walls with Modern Blast Retrofits: Experimental Results and Analytical Model. J. Struct. Eng..

[18] Natalia L.C., John J.M., Domenico A., Costantino M., Andrea P. (2018). Polyurea Coated and Plane Reinforced Concrete Panel Behavior under Blast Loading: Numerical Simulation to Experimental Results. Trends Civ. Eng. Archit..

[19] Amini M.R., Isaacs J.B., Nemat-Nasser S. (2010). Experimental Investigation of Response of Monolithic and Bilayer Plates to Impulsive Loads. Int. J. Impact Eng..

[20] Toutanji H.A., Choi H., Wong D., Gilbert J.A., Alldredge D.J. (2013). Applying a Polyurea Coating to High-Performance Organic Cementitious Materials. Constr. Build. Mater..

[21] Greene C.E., Myers J.J. (2013). Flexural and Shear Behavior of Reinforced Concrete Members Strengthened with a Discrete Fiber-Reinforced Polyurea System. J. Compos. Constr..

[22] Marawan A.E., Debaiky A.S., Khalil N.N. (2015). Shear and Flexural Behavior of RC Beams Strengthened with Polyurea Spray. Int. J. Adv. Res. Sci. Eng..

[23] Parniani S., Toutanji H. (2015). Monotonic and Fatigue Performance of RC Beams Strengthened with a Polyurea Coating System. Constr. Build. Mater..

[24] Carey N.L., Myers J.J. (2011). Discrete Fiber Reinforced Polyurea for Hazard Mitigation.

[25] Song J.-H., Lee E.-T., Eun H.-C. (2019). A Study on the Improvement of Structural Performance by Glass Fiber-Reinforced Polyurea (GFRPU) Reinforcement. Adv. Civ. Eng..

[26] Rizwan M., Khaleequzzaman S., uz Zaman U.K., Fida S.A., Shahzad A., Rehman M., Sulaiman M.O. (2021). Tensile Strength Improvement of Concrete Slabs Using Polyurea Spray. Pract. Period. Struct. Des. Constr..

[27] Szafran J., Matusiak A. (2020). Crushing Strength of Concrete Rings with a Polyurea Reinforce System. Tunn. Undergr. Space Technol..

[28] PPG VersaFlex|Arostruct^®^—VersaFlex. https://versaflex.com/products/industrial/arostruct-spray-polyurea/.

[29] Bridge Deck Membrane Technical Data Sheet. https://bridgepreservation.com/docs/prod/Data_BPbdm.pdf.

[30] Bridge Deck Membrane Topcoat Technical Data Sheet. https://bridgepreservation.com/docs/prod/Data_BPbdmtopcoat.pdf.

[31] VersaFlex|AquaVers-405 Technical Data Sheet. https://versaflex.com/wp-content/uploads/2020/11/TDS-AquaVers-405-072420-V.6.5.pdf.

[32] VersaFlex|SL-75 Technical Data Sheet. https://versaflex.com/wp-content/uploads/2020/11/SL75-TDS-080720-V.6.5.pdf.

[33] Nukote PP 300 TDS—Nukote Coating Systems. https://nukoteglobal.com/technical-sheets/nukote-pp-300-tds/.

[34] Nukote HTD TDS—Nukote Coating Systems. https://nukoteglobal.com/technical-sheets/nukote-htd-tds/.

[35] (2014). ASTM D638-14.

[36] Bischoff P.H., Perry S.H. (1991). Compressive Behaviour of Concrete at High Strain Rates. Mater. Struct..

[37] Li M., Li H. (2012). Effects of Strain Rate on Reinforced Concrete Structure under Seismic Loading. Adv. Struct. Eng..

[38] Philpot T.A. (2011). Mechanics of Materials: An Integrated Learning System.

